# Death of the Managing Editor’s Son

**Published:** 1887-02

**Authors:** 


### DEATH OF THE MANAGING EDITOR'S SON.

The readers of the SOUTHERN MEDICAL RECORD and the many friends of its Managing Editor, Prof. R. C. Word, M. D., will be pained to learn of his late great affliction. His gifted son, Hon. Ernest M. Word, died of typhlitis on the 11th of January, in the thirty-second year of his age. Hon. E. M. Word was a member of the State Senate, and, though the youngest member of that body, had acquired a reputation for ability, thorough devotion to his constituency, untiring energy and unflinching allegiance to honor and duty, which is seldom awarded to one so short a time in the political arena. The State loses a faithful servant, the XXXIVth District a most worthy and able champion, the family of Dr. Word a dutiful son and devoted brother.

The writer of this feels that he but echoes the sentiments of thousands of the medical profession in tendering to Professor Word and his bereaved family the tenderest sympathy of the medical fraternity at home and abroad. E. v. G.

